# Disparities in stage at diagnosis among breast cancer molecular subtypes in China

**DOI:** 10.1002/cam4.5792

**Published:** 2023-03-23

**Authors:** Hongmei Zeng, Siqi Wu, Fei Ma, John S. Ji, Lingeng Lu, Xianhui Ran, Jin Shi, Daojuan Li, Lan An, Rongshou Zheng, Siwei Zhang, Wanqing Chen, Wenqiang Wei, Yutong He, Jie He

**Affiliations:** ^1^ National Cancer Center/National Clinical Research Center for Cancer/Cancer Hospital Chinese Academy of Medical Sciences and Peking Union Medical College Beijing China; ^2^ The Fourth Hospital of Hebei Medical University and Hebei Tumor Hospital Shijiazhuang China; ^3^ Vanke School of Public Health Tsinghua University Beijing China; ^4^ Department of Chronic Disease Epidemiology Yale School of Public Health, Yale Cancer Center New Haven Connecticut USA

**Keywords:** breast cancer, China, molecular subtype, stage at diagnosis, surveillance, United States

## Abstract

**Background:**

Disease stage at diagnosis and molecular subtypes are the main determinants of breast cancer treatment strategies and prognosis. We aimed at examining the disparities and factors associated with the stage at diagnosis among the molecular subtypes in breast cancer patients in China.

**Methods:**

We identified patients with first primary breast cancer diagnosed between January 1, 2016, and December 31, 2017, from 23 hospitals in 12 provinces in China. We analyzed the proportion of non‐early‐stage (stages II–IV) breast cancer cases based on the family history of breast cancer, body mass index (BMI), insurance status, and molecular subtypes. Multivariable analyses were used to estimate the factors associated with non‐early‐stage diagnosis among the molecular subtypes. We further compared these estimates with that in the United States using the Surveillance, Epidemiology, and End Results database.

**Results:**

A total of 9398 Chinese were identified with first primary invasive breast cancer. Of the 8767 patients with known stages, the human epidermal growth factor receptor 2 (HER2)‐enriched subtype had the highest proportion of stages II–IV (76.6%) patients, followed by triple‐negative breast cancer (73.2%), luminal B (69.9%), and luminal A (62.3%). The percentage of non‐early‐stage patients was higher in women with overweight or obesity than in those with a body mass index (BMI) <25 kg/m^2^ (adjusted odds ratio [OR] 1.3, 95% confidence interval (CI) 1.1–1.4). Patients with a family history of breast cancer had a higher likelihood of early‐stage (adjusted OR 0.7, 0.5–0.8) breast cancer. Patients with rural insurance had a substantially higher risk of non‐early‐stage disease than those with urban insurance (adjusted OR 1.8, 1.4–2.2). Regarding the subtype, being overweight/obese only increased the risk of non‐early‐stage in luminal A breast cancer. Compared with the United States, China had a higher proportion of non‐early‐stage breast cancer for all subtypes, with the largest gap in luminal A (adjusted OR 2.2, 95% CI 2.0–2.4).

**Conclusion:**

The wide disparities in stage at breast cancer diagnosis imply that China urgently needs to improve early breast cancer diagnosis and health equity.

## Graphical abstract

This is the first study to analyze the stage distribution by breast cancer molecular subtype between China and the United States. Compared with the United States, China had a higher proportion of HER2‐enriched subtypes and more advanced stage for all subtypes. The wide disparities in breast cancer diagnosis imply an urgent need for improved breast cancer early detection and healthy equity in China.

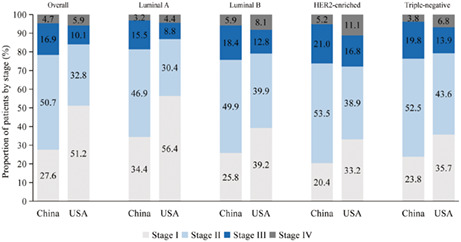

## 1 INTRODUCTION

Breast cancer is the most common malignancy among women globally, with an estimated 2.3 million new cases and 685,000 new deaths in 2020.^1^ Worldwide, China had the heaviest breast cancer burden in 2020, with 18.4% and 17.1% of newly diagnosed cases and deaths, respectively.^2^

Breast cancer is a heterogeneous disease with various molecular subtypes.^3^ Based on the presence of estrogen receptor (ER), progesterone receptor (PR), and human epidermal growth factor receptor (HER2), it can be categorized into four molecular subtypes: luminal A (ER^+^ and/or PR^+^, HER2^−^), luminal B (ER^+^ and/or PR^+^, HER2^+^), HER2‐enriched (ER^−^ and PR^−^, HER2^+^) and triple‐negative breast cancer (ER^−^ and PR^−^, HER2^−^).^4^ Molecular subtypes are one of the most important factors affecting prognosis.^5^ Luminal A tumors have the most favorable prognosis, partly because they are likely to benefit from hormonal therapy.^6^ Luminal B breast cancers have a more aggressive biology and are associated with worse prognosis.^7^ Targeted therapies against HER2 have substantially improved the outcomes of HER2‐enriched tumors.^8^ Triple‐negative breast cancer tends to grow and spread faster and has fewer treatment options among all the subtypes.^9^ In addition to subtypes, breast cancer survival also varies greatly by stage at diagnosis. Statistics from the American Cancer Society showed that the overall five‐year breast cancer survival rate was >99% for stage I and < 29% for stage IV.^10^ Our previous study on breast cancer in a single area of Beijing, China, also found that both overall and cancer‐specific survival rates were the lowest in patients with late stage at diagnosis.^11^

The importance of molecular subtypes in stage distribution has been well documented. Studies conducted in Malaysia and the United States, and our single area demonstrated that luminal A had a higher rate of early‐stage diagnosis than other molecular subtypes.^11^, ^12^, ^13^ Previous work by our group demonstrated that the proportion of stages II–IV accounted for 72.4% of breast cancers in China.^14^ However, stage distribution according to breast cancer molecular subtypes has not been reported using a large multicenter hospital database across different areas in China with various socioeconomic statuses. The United States has made significant progress in breast cancer control in the past decades, as represented by improvements in early detection and the overall decline in mortality.^10^ This article presents the stage distribution by molecular subtypes in breast cancer patients in China and compares it with the Surveillance, Epidemiology, and End Results (SEER) database covering an estimated 28% of the United States population.^15^ Understanding the stage disparities between the two countries may help improve breast cancer prognosis and narrow the survival gap.

The main purpose of our study was to describe the stage distribution of breast cancer molecular subtypes in the Chinese population and explore factors associated with non‐early‐stage based on the molecular subtypes. Our study assessed detailed information regarding stage at diagnosis according to the molecular subtypes and compared the data with that in the United States, which may provide scientific evidence for early detection, prognosis, and treatment of breast cancer.

## 2 METHODS

### 2.1 Study sample

In 2016, the National Cancer Center Registry (NCCR) launched a multicenter hospital‐based cancer registration program to collect information on patients' lifestyles and stages at diagnosis that were previously unavailable using population‐based cancer registries (Text S1). We selected 25 regions from 13 provinces located in six regions of China (east, middle, south, north, northeast, and northwest), reflecting different socioeconomic conditions and lifestyles (Table S1).

We analyzed data from hospital‐based NCCR in China and population‐based SEER Program in the United States for breast cancer patients diagnosed during 2016–2017, with detailed information on molecular subtypes and stage at diagnosis. We collected detailed demographic characteristics and clinical information from population‐based cancer registries and local hospitals. The inclusion criteria were as follows: (1) newly diagnosed during 2016–2017; (2) diagnosis and treatment at the abovementioned hospitals.

### 2.2 Data collection

Demographic characteristics, including place of residence, age at diagnosis, family history of breast cancer, body mass index (BMI), marital status, reproductive history, smoking history, and alcohol consumption, were collected by trained physicians via face‐to‐face interviews using a structured questionnaire. The place of residence refers to the registered residence at the time of breast cancer diagnosis. We used the area classification of the National Bureau of Statistics in China to categorize place of residence into urban/rural areas (Table S2). Family history refers to breast cancer in first‐ or higher‐degree relatives. Stages were routinely obtained from surgical reports, pathological reports, and imaging examinations performed by trained investigators at each hospital.

The variables were categorized as follows: age at diagnosis (< 55, 55–64, 65–74, and ≥ 75 years), place of residence (urban or rural), family history of breast cancer (no, yes), smoking history (no, yes), alcohol consumption (no, yes), and delivery history (no, yes). We characterized BMI into two categories. BMI ≥25.0 kg/m^2^ was classified as overweight/obesity.^16^ We divided the insurance status into three types: urban insurance, New Rural Cooperative Medical Care (NRCMSI), and others. The stage at diagnosis was classified based on the Manual of The American Joint Committee on Cancer Staging (7th edition).^17^ We defined early‐stage at diagnosis as stage I. Non‐early‐stage was defined as diagnosis at stages II–IV.

We extracted detailed information on the ER, PR, and HER2 status from pathological reports. The results of 1% or more positive nuclear staining of tumor were classified as positive ER (ER^+^) or PR (PR^+^).^18^ Positive HER2 (HER2^+^) was defined as positive nuclear staining intensity in “2+” and “3+” tumor cells.^19^ The molecular subtypes were classified as luminal A (ER^+^ and/or PR^+^, HER2−), luminal B (ER^+^ and/or PR^+^, HER2^+^), HER2‐enriched (ER^−^, PR^−^, HER2^+^), and triple‐negative breast cancer (ER^−^, PR^−^, HER2^−^).

### 2.3 Quality control

We conducted a series of trainings on the fundamentals of staging and coding to ensure data quality. Records were reviewed by the staff at each hospital. Data quality was further controlled after extraction. We used the International Agency for Research on Cancer (IARC)‐CanReg tools to evaluate the completeness and quality of data.^20^ A consistency check was performed, and if inconsistencies were found, the mistakes were corrected by retrieving the original records. The validity of the forms was checked by trained staff, and the data were entered into a data management system.

### 2.4 Data source from SEER in the United States

We obtained data from the SEER 18 registry database (2019 submission data) to compare stage distribution.^21^ Eligible breast cancer cases diagnosed between 2016 and 2017 were extracted using the SEER*Stat version 8.3.8. To ensure comparability, we used the same AJCC version 7th edition staging information from the SEER database. Cases in the United States were also classified into four molecular subtypes using the same immunohistochemical criteria as that used in Chinese patients.

### 2.5 Statistical Analyses

We presented the overall stage distribution of molecular subtypes based on family history, BMI status, and insurance status. Chi‐squared test was used to compare the association between candidate variables and stage distribution. We further explored the potential factors associated with non‐early‐stage for each breast cancer subtype. Multivariable logistic regression was used to estimate odds ratio (OR) and 95% confidence intervals (CIs). Stage analyses were adjusted for place of residence, age at diagnosis, BMI status, smoking history, alcohol consumption, reproductive history, family history of breast cancer, insurance status, hospital level, and hospital type. We compared the stage distribution based on molecular subtype between the two countries using binomial logistic regression. All analyses were performed using R software (version 4.1.2). All statistical tests were two‐sided, and *p* values less than 0.05 were considered statistically significant.

Several sensitivity analyses were conducted to verify the robustness of the results. First, we conducted logistic regression analysis with a different stage classification (stages I and II vs. stages III and IV) because studies used more stringent classifications to define the late stage.^22^ Second, we defined breast cancer molecular subtypes according to the 2013 St Gallen criteria.^23^

## 3 RESULTS

### 3.1 Stage distribution and baseline characteristics of Chinese patients

**FIGURE 1:**
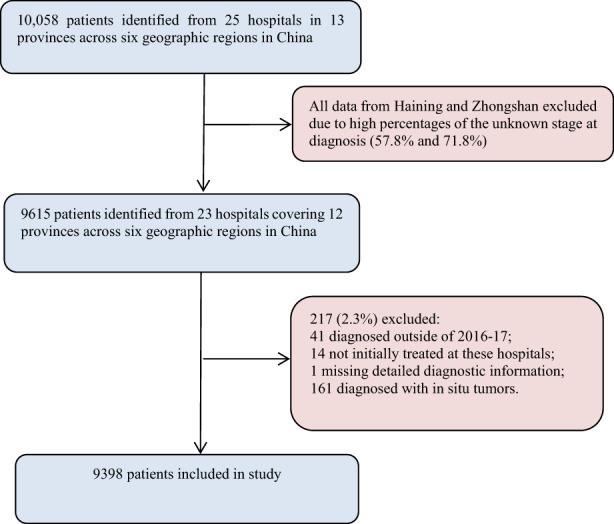
Flowchart of the study sample in China.

We included 9398 breast cancer patients from 23 hospitals in 12 provinces in China (Figure 1). As shown in Table 1, the largest patient group comprised those with luminal A (2628, 41.1%), followed by luminal B (2181, 34.1%), HER2‐enriched (883, 13.8%) and triple‐negative (704, 11.0%) breast cancer.

**TABLE 1:** Characteristics of Chinese breast cancer patients by molecular subtypes.

Characteristics	No. of Patients (%)					
	Overall	Luminal A	Luminal B	HER2‐enriched	Triple‐negative	Unknown
	(*n* = 9398)	(*n* = 2628)	(*n* = 2181)	(*n* = 883)	(*n* = 704)	(*n* = 3002)
Age at diagnosis (years)						
Mean (SD)	53.16 (11.3)	54.08 (11.6)	52.65 (11.1)	54.89 (10.1)	52.95 (11.4)	52.26 (11.3)
<55	5430 (57.8)	1415 (53.8)	1282 (58.8)	442 (50.1)	410 (58.2)	1881 (62.7)
55–64	2531 (26.9)	729 (27.7)	595 (27.3)	306 (34.7)	194 (27.6)	707 (23.6)
65–74	1066 (11.3)	345 (13.1)	236 (10.8)	107 (12.1)	70 (9.9)	308 (10.3)
≥75	371 (3.9)	139 (5.3)	68 (3.1)	28 (3.2)	30 (4.3)	106 (3.5)
Place of residence						
Urban	7528 (80.1)	2264 (86.1)	1796 (82.3)	731 (82.8)	580 (82.4)	2157 (71.9)
Rural	1870 (19.9)	364 (13.9)	385 (17.7)	152 (17.2)	124 (17.6)	845 (28.1)
BMI (kg/m^2^)						
<25.0	4976 (52.9)	1484 (56.5)	1318 (60.4)	555 (62.9)	424 (60.2)	1195 (39.8)
≥25.0	2435 (25.9)	900 (34.2)	608 (27.9)	222 (25.1)	190 (27.0)	515 (17.2)
Unknown	1987 (21.1)	244 (9.3)	255 (11.7)	106 (12.0)	90 (12.8)	1292 (43.0)
Marriage status						
Married	9036 (96.1)	2518 (95.8)	2089 (95.8)	854 (96.7)	670 (95.2)	2905 (96.8)
Other^a^	362 (3.9)	110 (4.2)	92 (4.2)	29 (3.3)	34 (4.8)	97 (3.2)
Unknown	10 (0.1)	1 (0.0)	1 (0.0)	0 (0.0)	4 (0.6)	4 (0.1)
Family history of breast cancer						
No	8794 (95.3)	2431 (94.4)	2013 (94.8)	828 (95.8)	642 (93.3)	2880 (96.8)
Yes	431 (4.7)	143 (5.6)	110 (5.2)	36 (4.2)	46 (6.7)	96 (3.2)
Unknown	173 (1.8)	54 (2.1)	58 (2.7)	19 (2.2)	16 (2.3)	26 (0.9)
Smoking history						
Ever	121 (1.3)	27 (1.1)	51 (2.5)	9 (1.1)	16 (2.3)	18 (0.6)
Never	9021 (98.7)	2533 (98.9)	2017 (97.5)	839 (98.9)	665 (97.7)	2967 (99.4)
Unknown	256 (2.7)	68 (2.6)	113 (5.2)	35 (4.0)	23 (3.3)	17 (0.6)
Alcohol consumption						
Ever	78 (0.8)	21 (0.8)	27 (1.2)	3 (0.3)	4 (0.6)	23 (0.8)
Never	9279 (99.2)	2599 (99.2)	2146 (98.8)	879 (99.7)	692 (99.4)	2963 (99.2)
Unknown	41 (0.4)	8 (0.3)	8 (0.4)	1 (0.1)	8 (1.1)	16 (0.5)
Reproductive history						
No	329 (3.6)	106 (4.1)	73 (3.4)	27 (3.1)	22 (3.2)	101 (3.4)
Yes	8896 (96.4)	2474 (95.9)	2076 (96.6)	839 (96.9)	667 (96.8)	2840 (96.6)
Unknown	173 (1.8)	48 (1.8)	32 (1.5)	17 (1.9)	15 (2.1)	61 (2.0)
Medical insurance^b^						
Urban insurance	5084 (54.1)	1752 (66.7)	1188 (54.5)	541 (61.3)	450 (63.9)	1153 (38.4)
NRCMSI	1560 (16.6)	258 (9.8)	294 (13.5)	100 (11.3)	84 (11.9)	824 (27.4)
Others	2754 (29.3)	618 (23.5)	699 (32.0)	242 (27.4)	170 (24.1)	1025 (34.1)
Stage at diagnosis						
I	2421 (25.8)	859 (32.7)	530 (24.3)	173 (19.6)	161 (22.9)	698 (23.3)
II	4449 (47.3)	1171 (44.6)	1026 (47.0)	454 (51.4)	355 (50.4)	1443 (48.1)
III	1481 (15.8)	386 (14.7)	378 (17.3)	178 (20.2)	134 (19.0)	405 (13.5)
IV	416 (4.4)	79 (3.0)	121 (5.5)	44 (5.0)	26 (3.7)	146 (4.9)
Unknown	631 (6.7)	133 (5.1)	126 (5.8)	34 (3.9)	28 (4.0)	310 (10.3)
Stage at diagnosis (complete dataset with full stage information)						
I	2421 (27.6)	859 (34.4)	530 (25.8)	173 (20.4)	161 (23.8)	698 (25.9)
II	4449 (50.7)	1171 (46.9)	1026 (49.9)	454 (53.5)	355 (52.5)	1443 (53.6)
III	1481 (16.9)	386 (15.5)	378 (18.4)	178 (21.0)	134 (19.8)	405 (15.0)
IV	416 (4.7)	79 (3.2)	121 (5.9)	44 (5.2)	26 (3.8)	146 (5.4)
Hospital type						
Specialized	8019 (85.3)	2412 (91.8)	1935 (88.7)	799 (90.5)	620 (88.1)	2253 (75.0)
General	1379 (14.7)	216 (8.2)	246 (11.3)	84 (9.5)	84(11.9)	749 (25.0)
Hospital level						
Tertiary	8541 (90.9)	2475(94.2)	2014 (92.3)	814(92.2)	646(91.8)	2592 (86.3)
Nontertiary	857 (9.1)	153 (5.8)	167(7.7)	69(7.8)	58(8.2)	410 (13.7)

Abbreviations: BMI, body mass index; NRCMSI, New Rural Cooperative Medical Scheme Insurance.

Breast cancer subtypes were classified as follows: Luminal A (ER^+^ and/or PR^+^, HER2^−^), Luminal B (ER^+^ and/or PR^+^, HER2+), HER2‐enriched (ER^−^, PR^−^, HER2^+^), triple‐negative breast cancer (ER^−^, PR^−^, HER2^−^).

^a^ Other: including the unmarried, divorced or widowed.

^b^ Urban insurance including urban employment‐based basic medical insurance and urban resident‐based basic medical insurance; others including uninsured, other insurance, or unknown.

**FIGURE 2:**
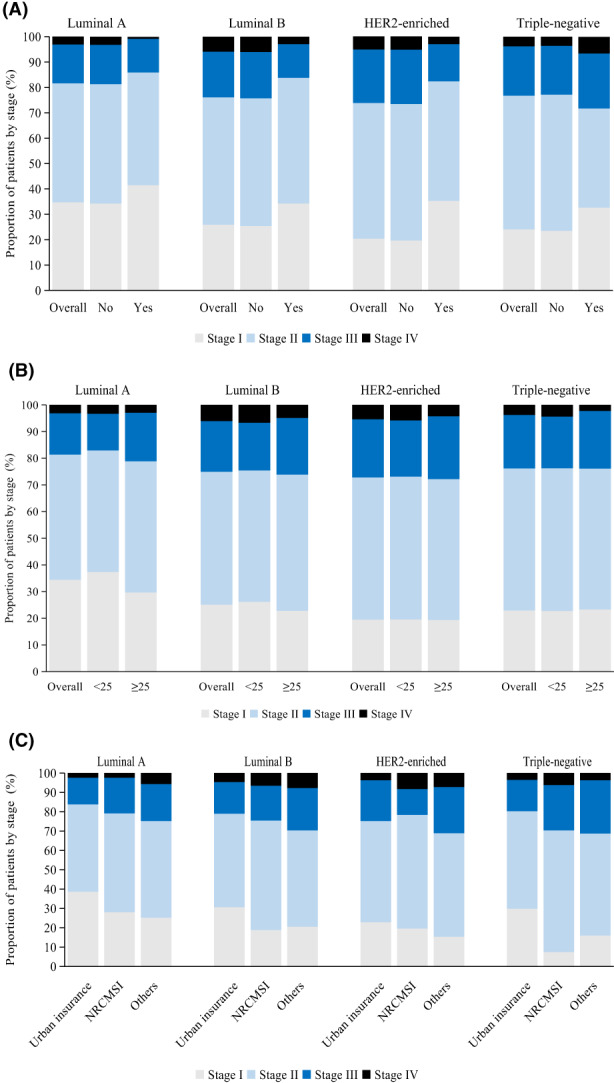
Stage distribution of breast cancer molecular subtypes by family history of breast cancer, body mass index(BMI), and medical insurance type. (A) By family history of breast cancer. (B) By BMI. (C) By medical insurance type.

Unknown stage accounted for 6.7% of all patients (*n* = 631; Table 1). Of the 8767 patients with known stage, 2421 (27.6%) had stage I, 4449 (50.7%) had stage II, 1481 (16.9%) had stage III, and 416 (4.7%) had stage IV disease. Of the 8767 patients, 6346 (72.4%) presented with stages II–IV. According to the molecular subtype, the proportion of patients with stages II–IV disease was highest for HER2‐enriched subtype (*n* = 676, 76.6%), followed by triple‐negative breast cancer (515, 73.2%), luminal B (1525, 69.9%) and luminal A (1636, 62.3%; Table 1, Figure 2).

### 3.2 Factors associated with non‐early‐stage based on the molecular subtype

The percentage of stages II–IV was higher in patients without than in those with a family history of breast cancer (72.7% vs. 63.1%; adjusted odds ratio [OR] 0.7, 95% CI 0.5–0.8). The disparity was particularly pronounced for non‐early‐stage luminal B breast cancer (adjusted OR 0.6, 0.4–0.9) and HER2‐enriched breast cancer (adjusted OR 0.4, 0.2–1.0; Figure 2A, Table 2), which occurred more frequently in patients without a family history. However, there was no difference between patients with or without a family history of non‐early‐stage luminal A or triple‐negative breast cancers.

**TABLE 2:** Unadjusted and adjusted odds ratios (ORs; 95% confidence intervals [CIs]) for stage II–IV disease according to molecular subtypes.

Factors	Unadjusted ORs (95% CIs)					Adjusted ORs (95% CIs)^a^				
	Overall	Luminal A	Luminal B	HER2‐enriched	Triple‐negative	Overall	Luminal A	Luminal B	HER2‐enriched	Triple‐negative
Place of residence										
Urban	1.0 (ref)	1.0 (ref)	1.0 (ref)	1.0 (ref)	1.0 (ref)	1.0 (ref)	1.0 (ref)	1.0 (ref)	1.0 (ref)	1.0 (ref)
Rural	1.0 (0.9–1.1)	0.9 (0.8–1.1)	0.9 (0.7–1.2)	1.0 (0.7–1.5)	0.8 (0.6–1.3)	1.1 (1.0–1.3)	1.2 (0.9–1.7)	0.9 (0.6–1.2)	1.2 (0.6–2.2)	1.3 (0.7–2.5)
Age at diagnosis										
<55	1.0 (ref)	1.0 (ref)	1.0 (ref)	1.0 (ref)	1.0 (ref)	1.0 (ref)	1.0 (ref)	1.0 (ref)	1.0 (ref)	1.0 (ref)
55–64	1.0 (0.9–1.1)	0.9 (0.8–1.1)	0.9 (0.7–1.2)	1.0 (0.7–1.5)	0.8 (0.6–1.3)	1.0 (0.9–1.1)	1.0 (0.8–1.2)	0.9 (0.7–1.1)	1.2 (0.8–1.8)	1.1 (0.7–1.8)
65–74	1.0 (0.8–1.1)	0.9 (0.7–1.1)	1.0 (0.7–1.4)	0.7 (0.4–1.2)	1.1 (0.6–2.0)	1.0 (0.8–1.1)	0.9 (0.7–1.2)	1.0 (0.7–1.4)	0.8 (0.4–1.4)	1.6 (0.8–3.2)
≥5	0.8 (0.6–1.0)	0.7 (0.5–1.0)	0.6 (0.4–1.1)	0.5 (0.2–1.1)	1.4 (0.5–3.8)	0.9 (0.7–1.1)	0.8 (0.5–1.2)	0.8 (0.4–1.5)	0.5 (0.2–1.2)	1.8 (0.6–5.6)
BMI (kg/m^2^)										
<25.0	1.0 (ref)	1.0 (ref)	1.0 (ref)	1.0 (ref)	1.0 (ref)	1.0 (ref)	1.0 (ref)	1.0 (ref)	1.0 (ref)	1.0 (ref)
≥25.0	1.2 (1.1–1.4)	1.4 (1.2–1.7)	1.2 (0.9–1.5)	1.0 (0.7–1.5)	1.0 (0.6–1.5)	1.3 (1.1–1.4)	1.5 (1.2–1.8)	1.3 (1.0–1.6)	1.0 (0.7–1.6)	1.1 (0.7–1.7)
Family history of breast cancer										
No	1.0 (ref)	1.0 (ref)	1.0 (ref)	1.0 (ref)	1.0 (ref)	1.0 (ref)	1.0 (ref)	1.0 (ref)	1.0 (ref)	1.0 (ref)
Yes	0.6 (0.5–0.8)	0.7 (0.5–1.1)	0.7 (0.4–1.0)	0.5 (0.2–0.9)	0.6 (0.3–1.2)	0.7 (0.5–0.8)	0.8 (0.5–1.1)	0.6 (0.4–0.9)	0.4 (0.2–1.0)	0.6 (0.3–1.2)
Smoking history										
Never	1.0 (ref)	1.0 (ref)	1.0 (ref)	1.0 (ref)	1.0 (ref)	1.0 (ref)	1.0 (ref)	1.0 (ref)	1.0 (ref)	1.0 (ref)
Ever	2.0 (1.2–3.2)	2.2 (0.8–5.8)	1.8 (0.8–3.8)	0.3 (0.1–1.5)	4.9 (0.6–37.4)	2.3 (1.3–4.1)	2.3 (0.8–7.3)	1.5 (0.6–4.0)	0.6 (0.1–4.7)	2.3 (0.3–21.8)
Alcohol consumption										
Never	1.0 (ref)	1.0 (ref)	1.0 (ref)	1.0 (ref)	1.0 (ref)	1.0 (ref)	1.0 (ref)	1.0 (ref)	1.0 (ref)	1.0 (ref)
Ever	1.6 (0.9–2.9)	2.7 (0.1–6.3)	2.6 (0.8–8.6)	0.1 (0.0–1.4)	‐	1.5 (0.8–3.0)	1.9 (0.5–1.2)	2.2 (0.6–7.8)	0.3 (0.0–6.4)	‐
Reproductive history										
No	1.0 (ref)	1.0 (ref)	1.0 (ref)	1.0 (ref)	1.0 (ref)	1.0 (ref)	1.0 (ref)	1.0 (ref)	1.0 (ref)	1.0 (ref)
Yes	1.2 (0.9–1.6)	1.0 (0.6–1.5)	0.7 (0.6–0.9)	1.5 (0.5–4.4)	1.9 (0.5–6.5)	1.3 (0.9–1.7)	1.0 (0.6–1.6)	0.8 (0.6–1.0)	1.5 (0.5–4.5)	2.7 (0.8–9.5)
Medical insurance										
Urban insurance	1.0 (ref)	1.0 (ref)	1.0 (ref)	1.0 (ref)	1.0 (ref)	1.0 (ref)	1.0 (ref)	1.0 (ref)	1.0 (ref)	1.0 (ref)
NRCMSI	1.8 (1.6–2.1)	1.6 (1.2–2.2)	1.9 (1.4–2.6)	1.2 (0.7–2.1)	2.9 (1.6–5.1)	1.8 (1.4–2.2)	1.4 (1.0–2.0)	1.9 (1.3–2.9)	1.3 (0.6–2.8)	3.9 (1.7–8.9)
Other	1.6 (1.4–1.8)	1.9 (1.5–2.3)	1.7 (1.4–2.1)	1.6 (1.1–2.5)	2.9 (1.6–5.3)	1.8 (1.6–2.1)	1.8 (1.4–2.2)	1.8 (1.4–2.4)	2.1 (1.2–3.6)	3.4 (1.5–7.5)

Abbreviations: BMI, body mass index; NRCMSI, New rural cooperative medical scheme insurance; OR, odds ratio.

^a^ Adjusted for hospital level, hospital type, age at diagnosis, BMI, place of residence, family history of breast cancer, smoking history, alcohol consumption, reproductive history and medical insurance type.

Overweight or obese patients were more likely to have non‐early‐stage cancer compared to those with BMI <25 kg/m^2^ (adjusted OR 1.3, 95% CI 1.1–1.4). However, the association between patient stage and BMI varied according to the molecular subtype. The proportion of non‐early‐stage patients was more predominant in luminal A patients who were overweight or obese than in those with BMI < 25 kg/m^2^ (adjusted OR 1.5, 1.2–1.8; Figure 2B, Table 2). However, there was no significant difference between patients with different BMI statuses in the non‐early‐stage prevalence of other molecular subtypes of breast cancer.

For all subtypes, cases with New Rural Cooperative Medical Insurance Scheme (NRCMSI; adjusted OR 1.8, 1.4–2.2) had a higher prevalence of non‐early‐stage than those with urban insurance. Regardless of molecular subtype, uninsured patients or those with other insurance consistently had increased odds of non‐early‐stage disease than patients with urban insurance. And the gap was especially striking for triple‐negative breast cancer (adjusted OR 3.4, 1.5–7.5), followed by HER2‐enriched breast cancer (adjusted OR 2.1, 1.2–3.6; Figure 2C, Table 2).

### 3.3 Comparison of breast cancer between the United States and China

**FIGURE 3:**
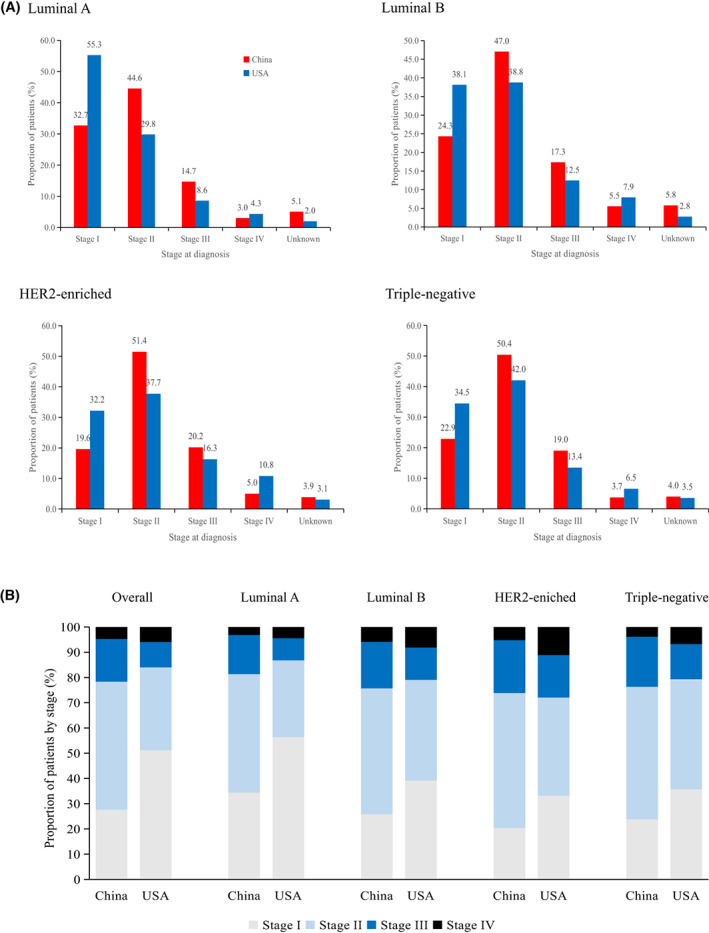
Stage distribution for breast cancer and by molecular subtype in China and in the United States. (A) Stage distribution for all patients. (B) Stage distribution for all patients excluded with unknown stage at diagnosis.

We included 134,514 patients with breast cancer from the SEER database, with detailed information on molecular subtypes and stage at diagnosis. Tables S3 and S4 reveal that patients in China had a higher percentage of luminal B and HER2‐enriched subtype than the Americans (*p* < 0.001). The prevalence of molecular subtypes in China and the United States was as follows: luminal A: 41.1% vs. 73.8%, luminal B: 34.1% vs. 11.2%, HER2‐enriched: 13.8% vs. 4.4% and triple‐negative breast cancer: 11.0% vs. 10.6%. Compared to those in the United States, patients in this study had a significantly higher proportion of stages II–IV and a higher incidence of unknown stages across all molecular subtypes (Figure 3, Table 3). For instance, the diagnostic gap between China and the United States for patients with stages II–IV was most prominent for luminal A (22%, OR 2.2, 95% CI 2.0–2.4), luminal B (13.4%; OR 1.8, 95% CI 1.6–2.0), HER2‐enriched (12.8%, OR 1.9, 95% CI 1.5–2.2) and triple‐negative breast cancer (11.9%, OR 1.6, 95% CI 1.3–1.9).

**TABLE 3:** Unadjusted and adjusted ORs (95% CIs) for stage II–IV breast cancer between China and the United States.

Factors	Unadjusted ORs (95% CIs)					Adjusted ORs (95% CIs)^a^				
	Overall	Luminal A	Luminal B	HER2‐enriched	Triple‐negative	Overall	Luminal A	Luminal B	HER2‐enriched	Triple‐negative
Age at diagnosis (years)										
<55	1.0 (ref)	1.0 (ref)	1.0 (ref)	1.0 (ref)	1.0 (ref)	1.0 (ref)	1.0 (ref)	1.0 (ref)	1.0 (ref)	1.0 (ref)
55–64	0.7(0.6–0.7)	0.7(0.7–0.7)	0.8(0.7–0.8)	0.8(0.7–0.9)	0.6(0.5–0.6)	0.7(0.7–0.7)	0.7(0.7–0.7)	0.8(0.7–0.8)	0.8(0.7–0.9)	0.6(0.5–0.6)
65–74	0.5(0.5–0.5)	0.5(0.5–0.6)	0.7(0.6–0.7)	0.6(0.5–0.7)	0.5(0.4–0.5)	0.5(0.5–0.6)	0.6(0.5–0.6)	0.7(0.6–0.8)	0.7(0.6–0.8)	0.5(0.4–0.5)
≥75	0.7(0.7–0.7)	0.7(0.7–0.8)	0.9(0.8–1.0)	0.7(0.6–0.8)	0.7(0.7–0.8)	0.8(0.7–0.8)	0.8(0.7–0.8)	0.9(0.8–1.0)	0.7(0.6–0.9)	0.7(0.7–0.8)
Country of residence										
United States	1.0 (ref)	1.0 (ref)	1.0 (ref)	1.0 (ref)	1.0 (ref)	1.0 (ref)	1.0 (ref)	1.0 (ref)	1.0 (ref)	1.0 (ref)
China	2.8 (2.6–2.9)	2.5 (2.3–2.7)	1.9 (1.7–2.1)	1.9 (1.6–2.3)	1.8 (1.5–2.1)	2.4 (2.3–2.6)	2.2 (2.0–2.4)	1.8 (1.6–2.0)	1.9 (1.5–2.2)	1.6 (1.3–1.9)

^a^ Adjusted for age group, country of residence.

In the sensitivity analyses, we did not find significant changes when defining non‐early‐stage as stages III and IV (Table S5). When classifying breast cancer molecular subtypes based on the 2013 St. Gallen criteria, the findings were similar to those of the main analysis (Table S6).

## 4 DISCUSSION

Understanding stage disparities according to molecular subtypes may provide scientific evidence for breast cancer prognosis and treatment. To our knowledge, this is the first report on stage distribution based on breast cancer molecular subtypes in a large multicenter hospital‐based dataset in China. We uncovered significant differences in the diagnostic stage of the molecular subtypes. In addition, we further explored the factors associated with non‐early‐stage disease and observed some disparities across breast cancer subtypes. Compared with the United States, China had a higher proportion of non‐early‐stage cases among all subtypes. These results are informative for understanding the pattern of breast cancer diagnosis in China. This study underscores the urgency to improve health equity in cancer detection and healthcare among rural residents in China.

Cancer outcomes vary widely between urban and rural areas in China.^24^ Difference in insurance status may be a potential cause of these disparities. Our study found that women who had no insurance tended to have non‐early breast cancer compared with women who had urban insurance, after adjusting for other factors. Previous studies conducted in the United States have shown that women with breast cancer who received Medicaid were diagnosed earlier than uninsured patients.^25^ Similarly, another study in the United States showed that Medicaid or uninsured patients get later‐diagnosis breast cancer than those with private insurance.^26^ Access to screening services may affect the breast cancer stage at diagnosis. In urban areas, residents often have better access to cancer diagnostics and treatment services.^27^, ^28^ For those living in rural areas, however, promoting the benefits of cancer screening and eradicating misconceptions about screening are priorities.^29^ For example, a study from the United States indicated that the cancer screening rate is lower among women in rural areas and communities if facilities for breast cancer screening services are not available.^30^ Therefore, public health interventions aimed at increasing facilities for cancer diagnosis and treatment in rural communities are urgently needed.

Previous studies have shown that overweight or obesity is associated with an increased risk of luminal tumors.^31^ In this study, we observed that women with luminal A breast cancer who were overweight/obese had a 50% higher risk of being diagnosed at a non‐early stage. Consistent with our results, studies found that women with BMI > 25.0 kg/m^2^ were 40%–70% more likely to be at a later stage than those with BMI < 25 kg/m^2^.^32^ In addition, a prospective cohort study indicated that in patients with luminal A breast cancer, obesity at diagnosis doubled the risk of death but did not influence the outcome for other subtypes.^33^ Our findings showed that patients with a family history had a higher rate of early diagnosis of luminal B and HER2‐enriched breast cancer. Similarly, a study from Geneva reported that a positive family history was positively associated with earlier breast cancer diagnosis, smaller tumors, and less lymph node involvement.^34^ Another large cohort study from China showed that patients without a family history of breast cancer were typically diagnosed at a later stage than those who had.^35^ Having a family history may prompt patients to be more aware of their health, resulting in the early detection of breast cancer. These results provide further evidence that differences in prognostic factors may exist between breast cancer molecular subtypes. Further studies are needed to determine the extent to which subtype‐specific factors influence survival disparities in breast cancer.

Compared with the United States, China had a consistently higher percentage of non‐early‐stage diagnoses for all molecular subtypes, with the largest diagnostic gap in the luminal A subtype. There may be several reasons for these disparities. First, high screening rates may influence the early detection of breast cancer. Since the early 1980s, biennial mammography has been available for women in the United States.^36^ In most provinces in China, screening for breast cancer is currently available, but the participation rates are still low due to hesitancy during health checkup toward cancer screening and low awareness among the target population for making informed choices.^29^ Second, the molecular subtypes of breast cancer differ among different races and ethnicities. We found that China had higher rates of luminal B, HER2‐enriched and triple‐negative breast cancers than the United States. Numerous studies have indicated that luminal A tumors are more common in white, Hispanic, and Asian populations, whereas triple‐negative breast cancer is more common in African populations.^37^ For example, the proportion of HER2‐enriched subtype in our study was much higher than the proportions previously reported for European countries and North America, which ranged from 4.5% to 6.4%, and was similar to that in other Asian countries (approximately 12.7%–21.5%).^38^

Our study has some limitations. First, this is an observational study on the stage at diagnosis in breast cancer molecular subtypes; whether the association between selected covariates and stage at diagnosis is a causal link needs to be explored in future studies. Second, this study cannot represent the overall Chinese population because of hospital‐based sources. The subjects of this study were breast cancer cases from multiple hospitals with different socioeconomic statuses across different areas of China. Considering that most patients were from urban areas with a high level of socioeconomic development, the proportion of patients with non‐early‐stage breast cancer in China could be higher than that observed in our results. Third, the evaluation of screening effect on stage migration was not possible in this study. Further studies are required to evaluate the longitudinal trends in stage distribution.

In conclusion, our study contributes to the understanding of stage disparities according to breast cancer molecular subtype. Expanding social medical insurance and improving medical facilities and cancer screening may narrow the gap in breast cancer survival between urban and rural areas. Future research is necessary to develop targeted interventions to improve breast cancer outcomes.

## AUTHOR CONTRIBUTIONS

**Hongmei Zeng:** Conceptualization (equal); funding acquisition (lead); project administration (lead); resources (lead); supervision (equal); validation (equal). **Siqi Wu:** Formal analysis (equal); validation (equal); visualization (equal); writing – original draft (equal); writing – review and editing (equal). **Fei Ma:** Conceptualization (equal); project administration (equal); resources (equal). **John S. Ji:** Supervision (equal). **Lingeng Lu:** Supervision (equal). **Xianhui Ran:** Data curation (equal); formal analysis (equal). **Jin Shi:** Investigation (equal); project administration (supporting). **Daojuan Li:** Project administration (supporting). **Lan An:** Data curation (supporting); project administration (supporting). **Rongshou Zheng**: Data curation (supporting); project administration (supporting). **Siwei Zhang**: Data curation (supporting); project administration (supporting). **Wanqing Chen:** Funding acquisition (lead); project administration (lead). **Wenqiang Wei:** Funding acquisition (lead); project administration (lead). **Yutong He:** Conceptualization (lead); data curation (lead); project administration (equal); supervision (equal); writing – review and editing (equal). **Jie He:** Funding acquisition (lead); project administration (equal).

## FUNDING INFORMATION

This work has received funding by grant from the Foundation for Innovative Research Groups of the National Natural Science Fund of China (grant number: 81672819).

## CONFLICT OF INTEREST STATEMENT

The authors declared no conflicts of interest.

## PATIENT CONSENT STATEMENT

Written signed consent was obtained from patient(s).

## ETHICS APPROVAL STATEMENT

This study was approved by the ethics committee of National Cancer Center/Cancer Hospital, Chinese Academy of Medical Sciences and Peking Union Medical College (approval number: No. 18–016/1645).

## Supporting information


Data S1

## Data Availability

The study group welcomes potential collaboration to maximise the use of data. A data dictionary, a detailed study protocol, and the R programmes can be reached by contacting the corresponding author of this paper. Due to Chinese legal restrictions and the current ethical approval for the study, data are not publicly available to share, but the research group can provide descriptive data in table form. Requests can be made to HZ.
