# Supplementary material for: Disparities in stage at diagnosis among breast cancer molecular subtypes in China
Source: Cancer Med. 2023 Mar 23;12(9):10865–76. doi: 10.1002/cam4.5792 (PMC10225199; doi:10.1002/cam4.5792)
Supplement: Supplementary file 1 — Data S1 [file CAM4-12-10865-s001.docx]

**Supplementary appendix**

Supplementary to: Zeng H, Wu S, Ma F, et al. Disparities in stage at diagnosis among breast cancer molecular subtypes in China

**Table of contents**

Text S1. The rationale and criteria for hospital selection

Table S1. The basic characteristics of 25 areas and hospitals in China during 2016-17

Table S2. Socioeconomic level during 2016-17 by area in China (mainland)

Table S3. Differences in characteristics of breast cancer patients between China and the United States

Table S4. Characteristics of breast cancer patients from SEER, stratified by molecular subtypes

Table S5. Adjusted odds ratios (ORs) (95% confidence intervals [CIs]) for stage Ⅲ-IV disease by molecular subtypes using data of patients with known stage

Table S6. Adjusted ORs (95% CIs) for stage II-IV disease using data of patients with known stage, using the St. Gallen criteria

**Text S1. The rationale and criteria for hospital selection**

The rationale for hospital inclusion was based on the following criteria.

1. Mainland China is classified into different geographical areas. Based on the geographical location and the distribution of population density of China, National Central Cancer Registry (NCCR) selected 13 provinces from six different geographical of China (2 in Northeast China, 2 in North China, 4 in East China,2 in Central China, 2 in South China, 1 in Northwest China).
2. Within 13 provinces, 25 counties/cities were chosen based on the following criteria: (1) Population-based cancer registration had been conducted in the selected area for more than five years. (2) The data quality of the population-based cancer registration is certified by International Agency for Research on Cancer/NCCR. (3) Death surveillance system and cancer follow-up system had been built up for more than five years so that continuous follow-up of the cancer patients would be feasible. (4) The population-based cancer registry had the capacity and willingness to conduct hospital-based cancer registration.
3. Given that most cancer patients would choose specialised or large general hospitals for cancer treatment, the largest specialised cancer hospital/general hospital within 25 areas were invited to be included. The selected hospital should have Electronic Medical Records and Health Information System to ensure accurate, efficient and complete information collection. If the largest specialised cancer hospital/general hospital did not have capacity/willingness to conduct hospital-based cancer registration, the second largest hospital would be chosen.

**Table S1. The basic characteristics of 25 areas and hospitals in China during 2016-17**

| **City/county** | **Region** | **Province** | **Disposable income per capita in the city/county (Yuan)** | **Area (urban/rural)** | **Hospital name** |
| --- | --- | --- | --- | --- | --- |
| Baoying | East China | Jiangsu | 22433 | rural | Baoying People’s Hospital |
| Beijing | North China | Beijing | 54880 | urban | Cancer Hospital of Chinese Academy of Medical Sciences |
| Cixian | North China | Hebei | 18658 | rural | Cixian Cancer Hospital |
| Feicheng | East China | Shandong | 25270 | rural | Feicheng People’s Hospital |
| Haining | East China | Zhejiang | 42739 | rural | Haining Cancer Hospital |
| Hangzhou | East China | Zhejiang | 47974 | urban | Zhejiang Cancer Hospital |
| Harbin | Northeast China | Heilongjiang | 24697 | urban | Heilongjiang Cancer hospital |
| Jiashan | East China | Zhejiang | 41412 | rural | Jiashan People’s Hospital |
| Jingshan | Central China | Hubei | 22796 | rural | Jingshan People's Hospital |
| Jingxian | East China | Anhui | 26199 | rural | Jingxian Hospital |
| Jintan | East China | Jiangsu | 34179 | rural | Jintan People’s Hospital |
| Linzhou | Central China | Henan | 22303 | rural | Linzhou Cancer Hospital |
| Luoshan | Central China | Henan | 17673 | rural | Luoshan People’s Hospital |
| Luoyang | Central China | Henan | 21824 | urban | The Third People’s Hospital of Luoyang |
| Nanning | South China | Guangxi | 23923 | urban | Affiliated Tumour Hospital of Guangxi Medical University |
| Qidong | East China | Jiangsu | 29920 | rural | Qidong People’s Hospital |
| Shenyang | Northeast China | Liaoning | 27600 | urban | Liaoning Cancer Hospital |
| Sheyang | East China | Jiangsu | 22735 | rural | Sheyang People’s Hospital |
| Shijiazhuang | North China | Hebei | 23652 | urban | The Fourth Hospital of Hebei Medical University |
| Tongling | East China | Anhui | 22283 | urban | Tongling People’s Hospital |
| Wuhan | Central China | Hubei | 37013 | urban | Hubei Cancer Hospital |
| Wuhu | East China | Anhui | 30668 | urban | The Second People’s Hospital of Wuhu |
| Xining | Northwest China | Qinghai | 22740 | urban | Qinghai Cancer Hospital |
| Zhengzhou | Central China | Henan | 29298 | urban | Henan Cancer Hospital |
| Zhongshan | South China | Guangdong | 41783 | urban | Zhongshan People’s Hospital |

**Table S2. Socioeconomic level during 2016-17 by area in China (mainland)**

|  | **Disposable income per capita (Yuan)^a^** | **Doctors per 1000^b^** | **Hospital beds per 1000^b^** |
| --- | --- | --- | --- |
| Overall mainland China | 24898 | 6.3 | 5.5 |
| Urban area | 35006 | 10.6 | 8.6 |
| Rural area | 12898 | 4.2 | 4.1 |

a: Data are from the National Bureau of Statistics of China

b: National Health Commission. China health statistical yearbook. China Union Medical University Press, 2019

**Table S3.** **Differences in characteristics of breast cancer patients between China and the United States**

| **Characteristics** | **China** | **United States** | ***P* value^a^** |
| --- | --- | --- | --- |
|  | (N = 9398) | (N = 134514) |  |
| **Age at diagnosis (years)** |  |  |  |
| <55 | 5430 (57.8) | 38875 (28.9) | <0.001 |
| 55-64 | 2531 (26.9) | 34708 (25.8) |  |
| 65-74 | 1066 (11.3) | 35756 (26.6) |  |
| ≥75 | 371 ( 3.9) | 25175 (18.7) |  |
| **Molecular subtye** |  |  |  |
| Luminal A | 2628 (28.0) | 92346 (68.7) | <0.001 |
| Luminal B | 2181 (23.2) | 13986 (10.4) |  |
| HER2-enriched | 883 ( 9.4) | 5570 (4.1) |  |
| Triple-negative | 704 ( 7.5) | 13292 (9.9) |  |
| Unknown | 3002 (31.9) | 9320 (6.9) |  |
| **Molecular subtye (complete dataset with full stubtype information)** |  |  |  |
| Luminal A | 2628 (41.1) | 92346 (73.8) | <0.001 |
| Luminal B | 2181 (34.1) | 13986 (11.2) |  |
| HER2-enriched | 883 ( 13.8) | 5570 (4.4) |  |
| Triple-negative | 704 ( 11.0) | 13292 (10.6) |  |
| **Stage at diagnosis** |  |  |  |
| Ⅰ | 2421 (25.8) | 66132 (51.2) | <0.001 |
| II | 4449 (47.3) | 42451 (32.8) |  |
| Ⅲ | 1481 (15.8) | 13003 (10.1) |  |
| Ⅳ | 416 ( 4.4) | 7659 ( 5.9) | <0.001 |
| Unknown | 631 ( 6.7) | 5269 ( 3.9) |  |

a. χ2 test

**Table S4. Characteristics of breast cancer patients from SEER, stratified by molecular subtypes**

| **Characteristics** | **No. of Patients (%)** | | | | | |
| --- | --- | --- | --- | --- | --- | --- |
|  | **Overall** | **Luminal A** | **Luminal B** | **HER2-enriched** | **Triple-negative** | **Unknown** |
|  | (N = 134514) | (N =13292) | (N =5570) | (N =92346) | (N =13986) | (N =9320) |
| **Age at diagnosis (years)** |  |  |  |  |  |  |
| <55 | 38875 (28.9) | 4725 (35.5) | 2140 (38.4) | 24272 (26.3) | 5426 (38.8) | 2312 (24.8) |
| 55-64 | 34708 (25.8) | 3447 (25.9) | 1579 (28.3) | 23728 (25.7) | 3757 (26.9) | 2197 (23.6) |
| 65-74 | 35756 (26.6) | 3084 (23.2) | 1126 (20.2) | 26397 (28.6) | 2936 (21.0) | 2213 (23.7) |
| ≥75 | 25175 (18.7) | 2036 (15.3) | 725 (13.0) | 17949 (19.4) | 1867 (13.3) | 2598 (27.9) |
| **Race** |  |  |  |  |  |  |
| White | 104212 (77.5) | 9250 (69.6) | 3960 (71.1) | 73564 (79.7) | 10507 (75.1) | 6931 (74.4) |
| Black | 15269 (11.4) | 2849 (21.4) | 800 (14.4) | 8826 ( 9.6) | 1717 (12.3) | 1077 (11.6) |
| Asian or Pacific Islander | 12549 ( 9.3) | 989 ( 7.4) | 688 (12.4) | 8496 ( 9.2) | 1538 (11.0) | 838 ( 9.0) |
| American Indian/Alaska Native | 815 ( 0.6) | 82 ( 0.6) | 45 ( 0.8) | 542 ( 0.6) | 84 ( 0.6) | 62 ( 0.7) |
| Unknown | 1669 ( 1.2) | 122 ( 0.9) | 77 ( 1.4) | 918 ( 1.0) | 140 ( 1.0) | 412 ( 4.4) |
| **Primary Site** |  |  |  |  |  |  |
| C50.0-Nipple | 466 ( 0.3) | 30 ( 0.2) | 38 ( 0.7) | 261 ( 0.3) | 51 ( 0.4) | 86 ( 0.9) |
| C50.1-Central portion of breast | 6252 ( 4.6) | 430 ( 3.2) | 283 ( 5.1) | 4419 ( 4.8) | 726 ( 5.2) | 394 ( 4.2) |
| C50.2-Upper-inner quadrant of breast | 16817 (12.5) | 1748 (13.2) | 615 (11.0) | 12041 (13.0) | 1639 (11.7) | 774 ( 8.3) |
| C50.3-Lower-inner quadrant of breast | 7169 ( 5.3) | 758 ( 5.7) | 295 ( 5.3) | 5018 ( 5.4) | 714 ( 5.1) | 384 ( 4.1) |
| C50.4-Upper-outer quadrant of breast | 45544 (33.9) | 5060 (38.1) | 1812 (32.5) | 31849 (34.5) | 4583 (32.8) | 2240 (24.0) |
| C50.5-Lower-outer quadrant of breast | 10384 ( 7.7) | 964 ( 7.3) | 432 ( 7.8) | 7284 ( 7.9) | 1151 ( 8.2) | 553 ( 5.9) |
| C50.6-Axillary tail of breast | 626 ( 0.5) | 84 ( 0.6) | 18 ( 0.3) | 415 ( 0.4) | 67 ( 0.5) | 42 ( 0.5) |
| C50.8-Overlapping lesion of breast | 30178 (22.4) | 2817 (21.2) | 1202 (21.6) | 21317 (23.1) | 3164 (22.6) | 1678 (18.0) |
| C50.9-Breast, NOS | 17078 (12.7) | 1401 (10.5) | 875 (15.7) | 9742 (10.5) | 1891 (13.5) | 3169 (34.0) |
| **Grade** |  |  |  |  |  |  |
| Well differentiated; Grade I | 29380 (21.8) | 280 ( 2.1) | 69 ( 1.2) | 27013 (29.3) | 797 ( 5.7) | 1221 (13.1) |
| Moderately differentiated; Grade II | 58560 (43.5) | 2385 (17.9) | 1333 (23.9) | 46338 (50.2) | 5943 (42.5) | 2561 (27.5) |
| Poorly differentiated; Grade III | 38013 (28.3) | 9951 (74.9) | 3718 (66.8) | 15991 (17.3) | 6526 (46.7) | 1827 (19.6) |
| Undifferentiated; Grade IV | 202 ( 0.2) | 46 ( 0.3) | 7 ( 0.1) | 44 ( 0.0) | 25 ( 0.2) | 80 ( 0.9) |
| Unknown | 8359 ( 6.2) | 630 ( 4.7) | 443 ( 8.0) | 2960 ( 3.2) | 695 ( 5.0) | 3631 (39.0) |
| **Tumor stage** |  |  |  |  |  |  |
| Ⅰ | 66132 (51.2) | 4580 (35.7) | 1791 (33.2) | 51024 (56.4) | 5331 (39.2) | 3406 (36.5) |
| II | 42451 (32.8) | 5587 (43.6) | 2101 (38.9) | 27536 (30.4) | 5420 (39.9) | 1807 (19.4) |
| Ⅲ | 13003 (10.1) | 1786 (13.9) | 906 (16.8) | 7929 ( 8.8) | 1742 (12.8) | 640 ( 6.9) |
| Ⅳ | 7659 ( 5.9) | 868 ( 6.8) | 601 (11.1) | 4005 ( 4.4) | 1108 ( 8.1) | 1077 (11.6) |
| Unknown | 5269 ( 3.9) | 471 ( 3.5) | 171 ( 3.1) | 1852 ( 2.0) | 385 ( 2.8) | 2390 (25.6) |

**Table S5. Adjusted ORs (95% confidence intervals [CIs]) for stage III–IV disease by molecular subtypes using data of patients with known stage**

| **Factors** | **Adjusted ORs (95% CIs)** | | | | |
| --- | --- | --- | --- | --- | --- |
|  | **Overall** | **Luminal A** | **Luminal B** | **HER2-enriched** | **Triple-negative** |
| **Age at diagnosis** |  |  |  |  |  |
| <55 | 1.0 (ref) | 1.0 (ref) | 1.0 (ref) | 1.0 (ref) | 1.0 (ref) |
| 55-64 | 0.9 (0.7-1.0) | 0.8 (0.6-1.1) | 0.9 (0.7-1.1) | 0.8 (0.5-1.2) | 1.0 (0.6-1.6) |
| 65-74 | 0.9 (0.7-1.1) | 0.8 (0.5-1.1) | 1.1 (0.8-1.6) | 1.4 (0.8-2.4) | 0.6 (0.3-1.4) |
| ≥75 | 1.0 (0.8-1.4) | 0.8 (0.5-1.4) | 1.0 (0.5-2.1) | 1.4 (0.6-3.4) | 1.7 (0.7-4.5) |
| **BMI* (kg/m^2^)** |  |  |  |  |  |
| <25.0 | 1.0 (ref) | 1.0 (ref) | 1.0 (ref) | 1.0 (ref) | 1.0 (ref) |
| ≥25.0 | 1.2 (1.0-1.3) | 1.4 (1.1-1.8) | 1.3 (1.0-1.6) | 1.1 (0.7-1.6) | 1.4 (0.9-2.2) |
| **Place of residence** |  |  |  |  |  |
| Urban | 1.0 (ref) | 1.0 (ref) | 1.0 (ref) | 1.0 (ref) | 1.0 (ref) |
| Rural | 1.2 (1.0-1.4) | 1.2 (0.9-1.8) | 1.1 (0.8-1.5) | 0.7 (0.4-1.2) | 1.3 (0.7-2.4) |
| **Family history of breast cancer** |  |  |  |  |  |
| No | 1.0 (ref) | 1.0 (ref) | 1.0 (ref) | 1.0 (ref) | 1.0 (ref) |
| Yes | 0.7 (0.5-1.0) | 0.8 (0.4-1.3) | 0.6 (0.3-1.1) | 0.7 (0.3-1.7) | 1.9 (0.9-3.9) |
| **Smoking history** |  |  |  |  |  |
| Never | 1.0 (ref) | 1.0 (ref) | 1.0 (ref) | 1.0 (ref) | 1.0 (ref) |
| Ever | 2.0 (1.3-3.2) | 2.5 (1.0-6.5) | 1.5 (0.6-3.3) | - | 1.7 (0.4-7.0) |
| **Alcohol consumption** |  |  |  |  |  |
| Never | 1.0 (ref) | 1.0 (ref) | 1.0 (ref) | 1.0 (ref) | 1.0 (ref) |
| Ever | 1.3 (0.7-2.4) | 0.7 (0.2-2.8) | 1.8 (0.7-4.5) | - | - |
| **Reproductive history** |  |  |  |  |  |
| No | 1.0 (ref) | 1.0 (ref) | 1.0 (ref) | 1.0 (ref) | 1.0 (ref) |
| Yes | 1.1 (0.8-1.5) | 1.4 (0.8-2.4) | 0.7 (0.6-0.9) | 1.4 (0.6-3.3) | 1.9 (0.7-5.2) |
| **Medical insurance** |  |  |  |  |  |
| Urban insurance | 1.0 (ref) | 1.0 (ref) | 1.0 (ref) | 1.0 (ref) | 1.0 (ref) |
| NRCMSI | 1.4 (1.1-1.7) | 1.4 (0.9-2.0) | 1.1 (0.7-1.6) | 1.2 (0.6-2.3) | 1.6 (0.8-2.9) |
| Others | 1.6 (1.4-1.9) | 1.7 (1.3-2.2) | 1.5 (1.2-2.0) | 2.0 (1.3-3.0) | 2.6 (1.4-4.6) |

*adjusted for hospital level, hospital type, age at diagnosis, BMI, place of residence, family history of breast cancer, smoking history, alcohol consumption, reproductive history and medical insurance type. BMI=body mass index. OR=odds ratio. NRCMSI, New rural cooperative medical scheme insurance

**Table S6. Adjusted ORs (95% CIs) for stage II–****IV disease using data of patients with known stage, using the St. Gallen criteria**

| **Factors** | **Adjusted ORs (95% CIs)** | | | | |
| --- | --- | --- | --- | --- | --- |
|  | **Overall** | **Luminal A** | **Luminal B** | **HER2-enriched** | **Triple-negative** |
| **Age at diagnosis** |  |  |  |  |  |
| <55 | 1.0 (ref) | 1.0 (ref) | 1.0 (ref) | 1.0 (ref) | 1.0 (ref) |
| 55-64 | 1.0 (0.9-1.2) | 0.9 (0.6-1.3) | 1.1 (0.9-1.4) | 1.0 (0.6-1.6) | 1.2 (0.8-1.9) |
| 65-74 | 1.0 (0.8-1.2) | 0.8 (0.5-1.2) | 1.2 (0.8-1.6) | 0.7 (0.3-1.4) | 1.5 (0.8-2.8) |
| ≥75 | 0.9 (0.7-1.1) | 0.7 (0.4-1.3) | 0.9 (0.5-1.5) | 0.4 (0.1-1.1) | 1.5 (0.5-4.3) |
| **BMI* (kg/m^2^)** |  |  |  |  |  |
| <25.0 | 1.0 (ref) | 1.0 (ref) | 1.0 (ref) | 1.0 (ref) | 1.0 (ref) |
| ≥25.0 | 1.3 (1.1-1.4) | 1.6 (1.2-2.1) | 1.2 (1.0-1.5) | 1.2 (0.7-1.9) | 1.1 (0.7-1.7) |
| **Place of residence** |  |  |  |  |  |
| Urban | 1.0 (ref) | 1.0 (ref) | 1.0 (ref) | 1.0 (ref) | 1.0 (ref) |
| Rural | 1.1 (1.0-1.3) | 1.1 (0.7-1.9) | 1.2 (0.9-1.6) | 1.5 (1.0-2.1) | 1.7 (0.8-3.4) |
| **Family history of breast cancer** |  |  |  |  |  |
| No | 1.0 (ref) | 1.0 (ref) | 1.0 (ref) | 1.0 (ref) | 1.0 (ref) |
| Yes | 0.7 (0.5-0.8) | 0.4 (0.2-0.8) | 0.8 (0.6-1.3) | 0.3 (0.1-0.7) | 0.6 (0.3-1.3) |
| **Smoking history** |  |  |  |  |  |
| Never | 1.0 (ref) | 1.0 (ref) | 1.0 (ref) | 1.0 (ref) | 1.0 (ref) |
| Ever | 2.0 (1.1-3.6) | 1.6 (0.3-9.1) | 3.5 (1.0-12.3) | - | 2.1 (0.2-18.9) |
| **Alcohol consumption** |  |  |  |  |  |
| Never | 1.0 (ref) | 1.0 (ref) | 1.0 (ref) | 1.0 (ref) | 1.0 (ref) |
| Ever | 1.5 (0.8-2.8) | - | 1.3 (0.4-4.2) | - | - |
| **Reproductive history** |  |  |  |  |  |
| No | 1.0 (ref) | 1.0 (ref) | 1.0 (ref) | 1.0 (ref) | 1.0 (ref) |
| Yes | 1.3 (0.9-1.7) | 1.0 (0.5-2.1) | 1.2 (0.7-1.9) | 1.4 (0.4-5.2) | 2.2 (0.7-6.8) |
| **Medical insurance** |  |  |  |  |  |
| Urban insurance | 1.0 (ref) | 1.0 (ref) | 1.0 (ref) | 1.0 (ref) | 1.0 (ref) |
| NRCMSI | 1.7 (1.4-2.1) | 1.0 (0.5-1.9) | 1.7 (1.2-2.4) | 1.3 (0.6-2.9) | 4.2 (1.4-12.5) |
| Others | 1.8 (1.6-2.1) | 1.6 (1.1-2.3) | 2.1 (1.7-2.7) | 2.3 (1.2-4.3) | 2.8 (1.6-4.9) |

*adjusted for hospital level, hospital type, age at diagnosis, BMI, place of residence, family history of breast cancer, smoking history, alcohol consumption, reproductive history and medical insurance. BMI=body mass index. OR=odds ratio. NRCMSI, New rural cooperative medical scheme insurance
